# Development and evaluation of adsorption sheet (HD safe sheet-U) using active carbon for the purpose of the preventing the contamination diffusion of urinary excreted anticancer drug

**DOI:** 10.1186/s40780-017-0085-8

**Published:** 2017-06-02

**Authors:** Junya Sato, Haruka Ohkubo, Yuki Sasaki, Makoto Yokoi, Yasunori Hotta, Kenzo Kudo

**Affiliations:** 1 Department of pharmacy, Iwate Medical University Hospital, 19-1 Uchimaru, Morioka, Iwate 020-8505 Japan; 2 Department of Clinical Pharmaceutics, School of Pharmacy, Iwate Medical University, 2-1-1 Nishitokuta, Yahaba, Iwate 028-3694 Japan; 3 Department of pharmacy, Shizuoka Cancer Center, 1007 Shimonagakubo, Nagaizumi-cho, Sunto-gun, Shizuoka, 411-8777 Japan; 4 Department of function group, Technology team, Futamura chemistry Co., Ltd., 2-29-16 Meieki, Nakamuraku, Nagoya, Aichi 450-0002 Japan

**Keywords:** Active carbon, Anticancer drug contamination, Adsorption sheet, HD safe sheet-U

## Abstract

**Background:**

Certain amount of anticancer drugs is excreted in the urine of patients receiving anticancer drugs, and urinary scattering including anticancer drugs at excretion has become a route of anticancer drug contamination. Therefore, we developed an active carbon sheet (HD safe sheet-U) that prevented diffusion by adsorbing anticancer drugs including that excreted in urine. The present study conducted a performance evaluation of this sheet.

**Methods:**

The adsorption performance of active carbon to anticancer drug in the urine was evaluated by determining concentration changes in the active carbon suspension (5 mg/mL) of 14 kinds of anticancer drugs (cyclophosphamide, ifosfamide, carboplatin, cisplatin, methotrexate, 5-fluorouracil, cytarabine, gemcitabine, doxorubicin, epirubicin, paclitaxel, docetaxel, etoposide, and irinotecan) diluted with artificial urine. Adhesion of the anticancer drug dropping on the sheet to a slipper sole was evaluated because urine including anticancer drugs is scattered on the floor, which can spread by adhering to shoe soles of patients and healthcare workers. The performance of the active carbon sheet was compared with two other types of medical adsorption sheets used as control sheets. Anticancer drugs diluted with artificial urine (1 mL) were dropped on the active carbon sheet and the two control sheets. The sheets were trod with slippers made by polyvinyl chloride. The adhered anticancer drug was wiped off and its quantity was determined.

**Results:**

A remarkable decrease in anticancer drug concentrations, except for cisplatin, was detected by mixture of active carbon in the artificial urine (0–79.6%). The quantity of anticancer drug adhesion to slipper soles from the active carbon sheet was significantly lower compared with that observed for the two control sheets for eight kinds of anticancer drugs (cyclophosphamide, ifosfamide, carboplatin, methotrexate, cytarabine, gemcitabine, doxorubicin, and docetaxel). There was no adhesion in cyclophosphamide and docetaxel. Furthermore, the quantities of adhesion in cytarabine, gemcitabine, doxorubicin, paclitaxel, and irinotecan were lower than determination limit.

**Conclusion:**

Active carbon might be effective in adsorbing urinary anticancer drugs. The active carbon sheet adsorbed urinary excreted anticancer drugs, and use of such sheets might prevent diffusion of contamination due to urinary excreted anticancer drugs.

**Electronic supplementary material:**

The online version of this article (doi:10.1186/s40780-017-0085-8) contains supplementary material, which is available to authorized users.

## Background

Anticancer drug exposure of healthcare workers and patients’ family members becomes health hazard risk. Although anticancer drug may be exposed in extremely small amounts compared with the amount administered to patients, studies have reported increased risk of acute toxicity, including diarrhea, cough, hair loss, exanthem [1–3], miscarriage as a reproductive toxicity [4], and carcinogenicity [5, 6]. Anticancer drugs are scattered during the processes of preparation, administration, disposal of administration equipment at the pharmacy and at patients’ bedside. These scattered anticancer drugs can be inhaled, come in contact with the skin, or are accidentally ingested by healthcare workers or patients’ family members (https://www.cdc.gov/niosh/docs/2004-165/pdfs/2004-165.pdf). The use of tools such as biological safety cabinet (BSC), closed system, and personal protection equipment (PPE) such as masks, gloves, and gowns are effective in protecting against exposure during the preparation and administration. Another contamination process is scattering of anticancer drugs through patients’ excrement. All body fluids such as urine, stool, vomit, saliva, sweat, and blood are considered patient excrement. In particular, urine is the most concerned excrement for diffusion because urine has a large volume and can easily scatter depending on the excretion posture, patient gender, and inappropriate handling during urine collection. Although urinary excretion of anticancer drugs may considerably vary by individual, most anticancer drugs are excreted in the unchanged form in the urine. The International Society of Oncology Pharmacy Practitioners guidelines recommend that PPE should be used during handling of excrement, including urine, within <48 h of administration (http://www.oncosystems.com.tr/dosyalar/ISOPP_Standards_of_ Practice-Safe Handling of Cy totoxics.pdf). In addition, for prevention of urinary scattering, the guidelines recommend the use of Western style toilet stool for urination for both men and women, sitting down during urination for men, and flushing after closing the cover of the toilet for patients receiving chemotherapy [7]. However, anticancer drug contamination of patients’ home and hospital lavatories has been reported. Yuki et al. surveyed anticancer drug contamination in home lavatories of patients with breast cancer [8]. Cyclophosphamide (CPA) at 0.04–8.35 ng/cm^2^ and 0.08–1.53 ng/cm^2^ were detected on the toilet seat and floor, respectively. These contamination levels are similar to those reported in various other contamination surveys conducted in hospital pharmacies [9, 10]. Moreover, Yuki et al. surveyed human exposure of family members who lived with patients receiving CPA administration [11]. CPA was detected at 17–252 ng per member in the urine in five members from 10 patient families. These findings suggested that healthcare workers and family members were more likely to be exposed to anticancer drugs owing to contact with patients’ body fluids including urine. Therefore, PPE should be used when healthcare workers and family members come in close contact with patients receiving chemotherapy. Morimoto et al. reported platinum contamination in a lavatory near an outpatient chemotherapy unit of a hospital [12]; platinum contamination of the floor of the toilet stool for women was <15–360 ng whereas that of the floor of the toilet stool for men was remarkably high (990–3000 ng). Urine may directly scatter on urination or scattered at a flash bulb. Moreover, anticancer drugs scattered on the lavatory floors adhered to slipper soles and seemed to spread outside the lavatory as well. Thus, when anticancer drug solution to be administered is scattered, use of a spill kit including PPE and inactivating agent is recommended to prevent contamination and diffusion while handling [13]. A wipe cleaning method using sodium hydroxide, sodium hypochlorite, and ozone waters is used for the preparation or administration environment, such as inside BSCs or hospital rooms where anticancer agent contamination occurs routinely [14–16]. However, this may lead to other problem such as damage to tiles, toilet stool, or other metal equipment, need for neutralization, and other uncertain degradation effects associated with the use of these inactivating agents in the lavatory. Such antiexposure methods for patients’ urine do not seem to be adequately performed in hospital and home settings. Therefore, a new method to prevent scattering of urinary anticancer drugs is warranted.

We developed active carbon sheets that adsorb the scattering splash during anticancer drug preparation (product name; HD safe sheet) [17]. Seven kinds of anticancer drugs diluted in a preparation concentration were dropped on an active carbon sheet; none of the drugs exhibited any adhesion to the infusion surface. In addition, the concentrations of these anticancer drugs were remarkably decreased in the active carbon suspension used for the active carbon sheet. We concluded that the active carbon sheet contributed to diffusive prevention of contamination by an active carbon adsorbing the anticancer drug scattered during preparation.

In the present study, we designed a concept to use this active carbon sheet for prevention of scattering of urinary anticancer drugs. First, we examined whether anticancer drugs were adsorbed by active carbon in the urine. Subsequently, we developed an improved version of active carbon sheet (product name; HD safe sheet-U) that enforced water absorptivity of a large volume of urine. We evaluated the diffusion prevention performance of urinary anticancer drugs with HD safe sheet-U.

## Methods

### Anticancer drug and artificial urine

The following anticancer drugs (pharmaceutical products) were used: cyclophosphamide hydrate (Endoxan® 500 mg, Shionogi & Co., Ltd. Osaka, Japan; CPA), ifosfamide (Ifomide® 1 g, Shionogi & Co., Ltd. Osaka, Japan; IFM), Carboplatin (Carboplatin 150 mg/15 mL, Pfizer Inc., Tokyo, Japan; CBDCA), cisplatin (Randa^Ⓡ^Inj 10 mg/20 mL, Nippon Kayaku Co., Ltd. Tokyo, Japan; CDDP), Methotrexate (Methotrexate® Injection 200 mg, Pfizer Inc., Tokyo, Japan; MTX), 5-fluorouracil (5-FU Injection 1000 mg, Kyowa Hakko Kirin Co. Ltd., Tokyo, Japan; 5-FU), cytarabine (Cylocide® N Injection 1 g/50 mL, Nippon Shinyaku Co. Ltd., Kyoto, Japan; Ara-C), gemcitabine hydrochloride (Gemcitabine I.V. infusion 200 mg [Yakult], Yakult Honsha Co. Ltd., Tokyo, Japan; GEM), doxorubicin hydrochloride (Doxorubicin Hydrochloride Injection 50 mg [NK], Nippon Kayaku Co. Ltd., Tokyo, Japan; ADR), epirubicin hydrochloride (Farmorubicin® RTU Inj 10 mg, Pfizer Inc., Tokyo, Japan; Epi-ADR), paclitaxel (Taxol Injection 30 mg, Bristol-Myers Squibb Company, Tokyo, Japan; PTX), docetaxel (Docetaxel Injection 20 mg/1 mL, Nippon Chemiphar Co. Ltd., Tokyo, Japan; DTX), etoposide (Etoposide Intravenous Infusion 100 mg [SANDOZ], Sandoz. Co. Ltd., Tokyo, Japan; VP-16), and irinotecan hydrochloride (Topotecin^Ⓡ^ Intravenous Drip Infusion 100 mg, Daiichi Sankyo Co. Ltd., Tokyo, Japan; CPT-11). These anticancer drugs were diluted with artificial urine or injectable distilled water.

The artificial urine was prepared according to Japanese Industrial Standard T3214 [18] by using the following composition and preparation method: urea 25.0 g, sodium chloride 9.0 g, anhydrous sodium dihydrogen phosphate 2.5 g, ammonium chloride 3.0 g, potassium dihydrogenphosphate (anhydride) 2.5 g, creatinine 2.0 g, and sodium sulfite (hydrate) 3.0 g were dissolved in 1.0 L of distilled water and regulated with phosphoric acid to achieve a pH of 6.6.

### Active carbon and HD safe sheet-U

Active carbon used in this study was prepared by Futamura Chemistry Co. Ltd. (Nagoya, Japan).

**Fig. 1:**
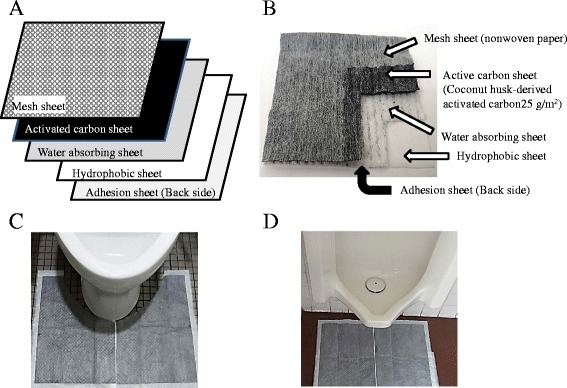
HD safe sheet-U (**a**: structure, **b**: photograph) and HD safe sheet-U application in lavatories (**c**: Western style toilet, **d**: Urinal toilet). Presents (**a**) structure and (**b**) photograph of HD safe sheet-U; (**c**, **d**) application of the sheet in lavatories. HD safe sheet-U consisted a nonwoven, adsorption layer that included active carbon at a density of 25 g/m^2^, a water (urine) absorption layer, an opacity film, and a back-side adhesive tape from the top

Materials for the active carbon were derived from a palm husk (average capillary diameter: 1.98 nm), with the following performance: specific surface area: 1,684 m^2^/g and iodine adsorption performance: 1,580 mg/g. The HD safe sheet-U including this active carbon was also prepared by Futamura Chemistry Co. Ltd. A photograph, structure, and directions for use of HD safe sheet-U were indicated in Fig. 1. Structurally, HD safe sheet-U consisted of a nonwoven, adsorption layer that included active carbon by a density of 25 g/m^2^, a water (urine) absorption layer, an opacity film, and a back-side adhesive tape from the top.

### Adsorptive evaluation of active carbon for urinary anticancer drug

Various anticancer drugs were diluted with artificial urine or distilled water following four series of diluted concentrations (CPA: 100–2,000 μg/mL, IFM: 20–1,000 μg/mL, CBDCA: 100–5,000 μg/mL, CDDP: 1–100 μg/mL, MTX: 50–6,000 μg/mL, 5-FU: 10–1,000 μg/mL, Ara-C: 50–1,000 μg/mL, GEM: 10–300 μg/mL, ADR: 50–1,000 μg/mL, Epi-ADR: 50–1,000 μg/mL, VP-16: 10–500 μg/mL, and CPT-11: 50–1,500 μg/mL). Active carbon was added to the anticancer drug solution at a concentration of 5 mg/mL. The mixture was shaken by a warm bath maintained at 25 °C for 1 h and centrifuged (20,800 *g* for 5 min). Anticancer drug concentrations were determined in the supernatant. For control, a mixture that did not include active carbon was treated under the same conditions and anticancer drug concentrations were determined in the supernatant.

### Evaluation of anticancer drug diffusive prevention performance

The diffusive prevention performance of urinary anticancer drug scattered on the sheet was evaluated based on adhesion of anticancer drugs to slippers soles from the sheet with dropped anticancer drugs. The dropping concentration of the anticancer drug set higher than urinary unchanged drug concentration. Unchanged anticancer drug concentrations in the urine were calculated as dose × urinary unchanged drug excretion rate ÷ urine volume. The dose was calculated with coverage dose by insurance in the Japanese normal body weight or surface area (58 kg or 1.73 m^2^) [19, 20]. The urinary unchanged drug excretion rate was referred from a value described in the package insert of each pharmaceutical product. The daily mean urine volume was assumed as 1,730 mL/1.73 m^2^ [21]. Table 1 summarizes the estimated urinary unchanged drug excretion concentrations and the dropping concentration per sheet of each anticancer drug. A comparison with HD safe sheet-U was performed with two medical absorbing sheets (control sheet 1: Pitapa sheet^Ⓡ^, Vilene Create Co. Ltd., Tokyo, Japan; control sheet 2: Absocare sheet, Lily Co. Ltd., Niigata, Japan). Three types of sheets were cut for 10 cm in every direction, and 1 mL of anticancer drug diluted with artificial urine was dropped on the sheets. Subsequently, an experimenter (body weight: 50 kg) stepped on the sheet in slippers made from vinyl chloride for 10 s. The anticancer drug that adhered to the slipper soles was wiped off using 5 × 5 cm of cotton and 5 mL of distilled water, and the wiped off recovery solution was centrifuged (20,800 *g*, 5 min). An anticancer drug concentration of the supernatant was determined. An anticancer drug adhesion to the slipper sole without using any sheet was determined as follows. An anticancer drug was dropped on a stainless steel bat directly. The stainless steel bat was stepped on with slippers. The drug that adhered to the slipper sole was wiped off and its concentration was determined. The recovery rate of this wipe off method was assessed by dropping an anticancer drug directly on the slipper sole. The anticancer drug on the slipper sole was wiped off and its concentration was determined.

**Table 1:** Urinary excretion concentration of the anticancer drug and dripping concentration

	Anticancer drug	Dosage^a^ (min ~ max)	Urinary unchanged drug excretion rate (% of dosage/24h)^b^	Urinary unchanged drug concentration^c^ (μg/mL)	Dripping concentration (μg/mL)
Alkylating drug	CPA	500mg/m^2^ – 60mg/kg	10.0	50.0 – 201.2	2,000
	IFM	0.8 – 3g/m^2^	6.0	48.0 – 180.0	1,000
Platinum drug	CBDCA	300 – 635mg/m^2^	57.0 – 82.0	171.0 – 520.7	5,000
	CDDP	10 – 100mg/m^2^	14.0 – 54.0	1.4 – 54.0	100
Antimetabolite drug	MTX	10mg/body – 300mg/kg	75.0 – 98.0(72h)	1.4 – 3285.5	6,000
	5-FU	5mg/kg – 2600mg/m^2^	10.0	16.8 – 260	1,000
	Ara-C	0.8mg/kg – 3,000mg/m^2^	7.1 – 7.8	1.9 – 234	1,000
	GEM	1,000 – 1,250mg/m^2^	5.3	53.0 – 66.3	300
Anthracycline drug	ADR	0.2mg/kg – 80mg/m^2^	11.5	0.8 – 9.2	1,000
	Epi-ADR	15 – 100mg/m^2^	6.2 (48h)	0.5 – 3.0	1,000
Taxane drug	PTX	80 – 210mg/m^2^	7.3 – 11.3(72h)	1.9 – 7.9	200
	DTX	60 – 75mg/m^2^	1.7 – 4.2 (48h)	0.5 – 1.6	200
Topoisomerase inhibitor	VP-16	60 – 500mg/m^2^	10.3 – 35.2	6.2 – 176.0	500
	CPT-11	20 – 180mg/m^2^	16.3 – 21.1	3.3 – 38.0	1,500

^a^; Insurance application dose in Japan, ^b^; Report value in each pharmaceutical products IF, ^c^;Dosege × Japanease standard body surface area (1.73m^2^) or body weight (58kg) × urinary unchanged drug excretion rate ÷standard urine volume (1,730mL/day)

### Anticancer drug determination

CBDCA and CDDP concentrations were determined using an atomic absorption photometer (AA-7000, Shimadzu Co. Ltd., Kyoto, Japan). As determination condition, an absorptivity of a wavelength of 265.9 nm due to a platinum atom ionized by the furnace method was detected. A standard curve was formed using a platinum standard solution (Kanto Chemical Co. Inc., Tokyo, Japan), and the determination limit was 0.05 μg/mL. Anticancer drugs except CBDCA and CDDP were determined using high-performance liquid chromatography with ultraviolet absorptiometry (D-2000 Elite System, Hitachi High-Technologies Co. Ltd., Tokyo, Japan). An octadecyl silicagel column (YMC-Pack ODS-A, 5 μm, 6 × 150 nm, YMC Co. Ltd., Kyoto, Japan) was used for determination. The measure method of these anticancer drugs used the method of mention for Japanese Pharmacopoeia or product interview form, previous article. The details of determination condition were shown in Table 2. The standard curve in each measurement set 6–8 concentrations. We used standard curve with straightness coefficient of correlation (r^2^) = 0.99 or more. The reproducibility of the measurement including within-day and day-to-day was less than ±5% of setting concentrations.

**Table 2:** HPLC-UV analysis condition

Anticancer drug	Column Temperature (°C)	Mobile phase (Volume%)	Flow rate (mL/min)	Detection Wavelength (nm)	Determination limit (μg/mL)
CPA	40	H_2_O : CH_3_CN = 75 : 25	l.0	195	5
IFM	40	H_2_O : CH_3_CN = 54 : 46	1.0	198	1.0
MTX	40	0.1%CH_3_COOH:CH_3_OH = 80:20	l.0	307	0.1
5-FU	25	10mM KH_2_PO_4_ :CH_3_OH = 90 : 10	l.0	285	0.75
Ara-C	40	50mMPBS(pH6.8):CH_3_OH = 99.5:0.5	1.0	275	0.1
GEM	40	10mMPBS(pH7.5):CH_3_OH = 98:2	1.0	275	0.1
ADR	40	90% CH_3_CN:50mM PBS (pH3.2) = 68 : 32	1.0	230	1.0
Epi-ADR	40	20mM PBS (pH2.0) : CH_3_CN = 69 : 31	l.0	254	0.2
PTX	40	CH_3_CN : H_2_O : CH_3_OH = 50 : 30 : 20	l.0	236	0.5
DTX	40	10mMPBS(pH3.0):CH_3_CN = 50:50	1.0	230	0.1
VP-16	40	0.007%TEA, 20mMPBS (pH5.2):CH3CN:CH3OH = 63:19:18	1.0	285	0.1
CPT-11	40	50mM KH_2_PO_4_, 7.5mM Bu_4_NBr : CH_3_CN = 83 : 17	l.0	254	0.5

*PBS* phosphate buffer saline, *Bu*_*4*_ *NBr* Tetrabutylammonium bromide, *TEA* triethlamine

### Statistics

Four sample preparations were conducted for both experiments. Each value was provided as mean ± standard deviation. For adsorption performance to the active carbon of urinary anticancer drug, the anticancer drug residual ratio for setting concentrations was compared. For comparing diffusive prevention performance of urinary anticancer drug scattered on the sheet, one-way analyses of variance (ANOVA) was performed; when this was significant, multiple comparisons were performed between the three sheets (Fisher’s least significant difference test). A hazard ratio of <5% represented a statistically significant difference. Statistical analysis was performed using Excel statistics 2012 (Social Survey Research Information, Co. Ltd., Tokyo, Japan).

## Results

### Adsorptive evaluation of active carbon to urinary anticancer drug

**Fig. 2:**
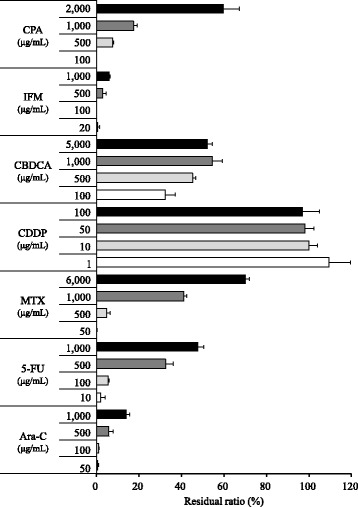
Residual ratio of anticancer drugs (CPA, IFM, CBDCA, CDDP, MTX, 5-FU, and Ara-C) in active carbon suspension. Bar indicates residual ratio for setting concentrations as mean ± standard deviation (%) (*n* = 4)

**Fig. 3:**
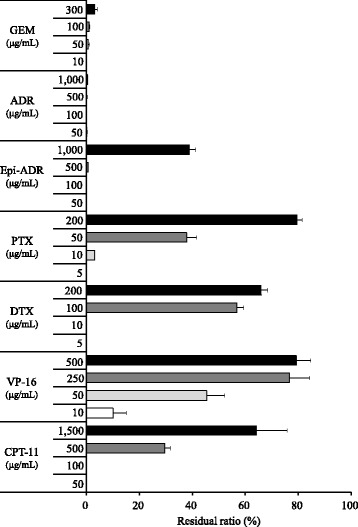
Residual ratio of anticancer drugs (GEM, ADR, Epi-ADR, PTX, DTX, VP-16, and CPT-11) in active carbon suspension. Bar indicates residual ratio for setting concentrations as mean ± standard deviation (%) (*n* = 4)

The adsorption performance of active carbon to urinary anticancer drug was indicated as a residual ratio after mixture with active carbon, as indicated in Figs. 2 and 3. All anticancer drugs except CDDP showed a tendency toward decrease of the residual rate as the concentrations lowered (0–79.6%). The changes in anticancer drug concentrations by mixture of artificial urine or water alone were not as remarkable when compared with mixture of active carbon (Additional file 1: Appendix data 1 and 2). The change in residual rates of most drug concentrations were less than ±25%, and the anticancer drug that showed the most exceptional decrease in this rate was 100 μg/mL of CPA diluted with urine (−36.1%). Moreover, the influence of artificial urine on adsorption performance of active carbon was not remarkable. The decrease of adsorptive performance by urine in most drug concentrations was <20%, and the difference of −31.7% in the residual rate was maximum exceptionally in 100 μg/mL of DTX.

**Fig. 4:**
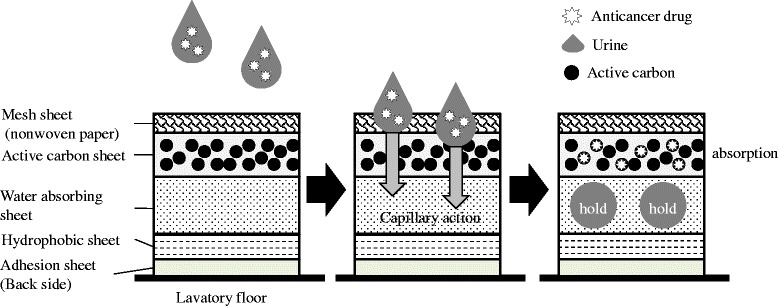
Mechanism by which HD safe seat-U adsorbs urine and urinary anticancer drugs. Indicates the assumed mechanism of action of HD safe sheet-U. The scattered urine passes through a mesh sheet and is aspirated to an active carbon bed, absorbing a water layer by the capillary phenomenon. The urinary anticancer drug molecules coming in contact with active carbon during the aspiration process will be adsorbed by active carbon. The anticancer drug once adsorbed by active carbon is isolated from slipper soles by using a mesh sheet

### Evaluation of anticancer drug diffusive prevention performance

The wipe-off recovery rates of the anticancer drugs that adhered to slipper soles were summarized in Table 3. Wipe-off recovery rates of all anticancer drugs except CDDP, Ara-C, Epi-ADR, and VP-16 were >80%. The coefficient of variation of the wipe-off recovery rate was <20% in each anticancer drug. Table 4 summarizes the quantity of anticancer drug adhering to slipper soles from each sheet of the anticancer drug dropped. The anticancer drug adhesion quantity of HD safe sheet-U was significantly lower than that of control sheet 1 for CPA, IFM, CBDCA, CDDP, MTX, Ara-C, GEM, ADR, DTX, and VP-16. Moreover, the anticancer drug adhesion quantity of HD safe sheet-U was significantly lower than that of control sheet 2 for CPA, IFM, CBDCA, MTX, Ara-C, GEM, ADR, and DTX. However, the anticancer drug adhesion quantity of HD safe sheet-U was not significantly lower than those of the two control sheets for 5-FU and Epi-ADR. Furthermore, the anticancer drug adhesion quantity of HD safe sheet-U and both control sheets was less than the determination limit (<2.5 μg) for PTX and CPT-11.

**Table 3:** Wipe off recovery of dropped anticancer drug from the slipper sole

Anticancer drug	Dropped amount (μg)	Recovery (μg)	Recovery (%)	CV (%)
CPA	2000	1947.2 ± 261.5	97.4 ± 13.1	13.4
IFM	1000	1094.6 ± 87.8	109.5 ± 8.8	8.0
CBDCA	5000	4240.5 ± 618.2	84.8 ± 12.4	14.6
CDDP	100	68.2 ± 11.5	68.2 ± 11.5	16.9
MTX	6000	4890.6 ± 413.3	81.5 ± 6.9	8.5
5-FU	1000	873.2 ± 33.4	87.3 ± 3.3	3.8
Ara-C	1000	726.3 ± 117.3	72.6 ± 11.7	16.1
GEM	300	300.4 ± 27.3	100.1 ± 9.1	9.1
ADR	1000	899.4 ± 121.1	89.9 ± 12.1	13.5
Epi-ADR	1000	475.5 ± 40.8	47.6 ± 4.1	8.6
PTX	200	200.0 ± 18.9	100.0 ± 9.5	9.5
DTX	200	171.1 ± 14.9	85.5 ± 7.4	8.7
VP-16	500	308.7 ± 46.8	61.7 ± 9.4	15.2
CPT-11	1500	1213.2 ± 159.5	80.9 ± 10.6	13.1

Mean ± SD (μg)(*n* = 4), *CV* coefficient of variation (%)

**Table 4:** Adhesion to the slipper sole of urinary anticancer drug dropped on the sheet

Anticancer drug	Dropping amount (μg)	No sheet	Control sheet 1	Control sheet 2	HD safe Sheet-U
CPA	2000	367.8 ± 16.3	355.3 ± 59.0	256.3 ± 44.3^**^	ND
IFM	1000	142.4 ± 26.8	32.5 ± 3.3	21.2 ± 2.2^***^	16.8 ± 2.1^*** †^
CBDCA	5000	275.2 ± 56.7	16.1 ± 5.0	11.9 ± 3.7	1.5 ± 0.5^*** ††^
CDDP	100	0.7 ± 0.2	0.3 ± 0.1	0.3 ± 0.1	0.2 ± 0.1^*^
MTX	6000	1183.1 ± 130.6	78.0 ± 6.4	53.6 ± 7.8^***^	5.0 ± 1.6^*** †††^
5-FU	1000	88.1 ± 9.1	5.1 ± 1.3	4.0 ± 0.3	3.8 ± 0.1
Ara-C	1000	137.4 ± 51.5	45.3 ± 15.8	3.1 ± 1.1^***^	LOQ
GEM	300	45.8 ± 8.5	27.4 ± 5.4	1.5 ± 0.1	LOQ
ADR	1000	198.7 ± 30.9	25.9 ± 3.7	7.7 ± 1.6^***^	LOQ
Epi-ADR	1000	8.8 ± 0.8	0.7 ± 0.2	0.9 ± 0.2^***^	0.6 ± 0.2
PTX	200	27.6 ± 9.5	LOQ	LOQ	LOQ
DTX	200	34.2 ± 7.1	2.7 ± 1.8	0.5 ± 0.0^*^	ND
VP-16	500	118.2 ± 29.5	3.4 ± 1.4	1.5 ± 0.7^*^	0.6 ± 0.1^*^
CPT-11	1500	63.9 ± 34.5	LOQ	LOQ	LOQ

The numerical value indicated mean ± standard deviation (μg)(*n* = 4), **P < 0.01, ***P < 0.001 vs. Control sheet 1, ^††^ *P* < 0.01, ^†††^ *P* < 0.001 vs. Control sheet 2 by Fisher’s least significant difference test. *LOQ* limit of quantitation (Ara-C; <0.5μg, GEM; <0.5μg, ADR;<5.0μg, PTX; <2.5μg, CPT-11; <2.5μg). *ND* No peak detection

## Discussion

As for the condition not to lay all sheets, all anticancer drugs attached to the slippers sole with remarkable quantity. Therefore, it seemed to be important for preventing diffusion of the contamination by laying an absorbent sheet around the toilet stool. HD safe sheet-U, developed in this study, immediately absorbs scattered urine, and active carbon adsorbs the urinary excreted anticancer drug. Moreover, adhesion quantity of an anticancer drug from slipper soles trod on the HD safe sheet-U was lower than that of both or one of the existing sheet for 10 of the 14 anticancer drugs used in this study. From these results, the adhesion prevention performance to the slippers sole of the scattered anticancer drug of the HD safe sheet-U seemed to be superior to an existing control sheets. A decrease in anticancer drug concentrations for most anticancer drugs except CDDP was found by mixing the drug with active carbon in artificial urine (−20.4 – −99.7%) even at comparatively higher concentrations of the anticancer drug. Based on these results, adsorption of active carbon seemed successful in inducing a decrease in anticancer drug adhesion from anticancer drugs dropped on the sheet. In urine, the solute including urea and creatinine etc is included for approximately 5%. However, adsorption was noted even when an anticancer drug was diluted with artificial urine. The urinary influence on anticancer drug adsorption performance of active carbon did not seem to have a considerable problem.

In this study, there was a difference in adsorption ability to HD safe sheet-U depending on the kind of the anticancer drug. As this reason, the adsorption mechanism of the active carbon, molecular weight and the polarity of the anticancer drug were thought to affect it. Generally, the ability for adsorption of the active carbon was caused by van der waals power on hydrophobic surface of the active carbon, and target molecules was attracted and adsorbed physically. The active carbon has a large number of the capillary called mesoporous and microporous, which make large contact area to target molecules. It is also important for adsorption by the active carbon that target particle diameter is smaller than these capillary because the target particle must get into the capillary porous. A material with nonpolar, and insolubility in water is easy to adsorb comparatively in aqueous solutions. Therefore, it was thought about adsorptive of ionized CDDP in distilled water is poor while being a small molecule.

The difference in anticancer drug adhesion quantity between the sheets was thought to be caused by the following material and structural differences in the three types of sheets other than the adsorption ability with active carbon. Control sheet 1 was a sheet for the purpose of preventing floor contamination and fall by liquids such as blood, body fluids, and contaminated water scattered in a hospital environment. Control sheet 1 constituted a laminating waterproofing resin and a water-absorptive, nonwoven, and adhesive back side. It was the thinnest with favorable ease of convenience. However, it was not able to often absorb 1 mL of artificial urine in the present study conditions. The prevention performance of anticancer drug diffusion from scattered urine in control sheet 1 did not seem adequate. Control sheet 2 was a medical sheet for the purpose of preventing body fluid diffusion during surgery and wound care. Control sheet 2 had three laminar structures consisting of nonwoven, absorbing pulp, and an impermeability film. Control sheet 2 rapidly absorbed 1 mL of artificial urine; however, adhesion of anticancer drugs to slipper soles was observed for all anticancer drugs. The structure of HD safe sheet-U comprised an active carbon bed and superabsorbent polymer for the purpose of adsorption of urinary anticancer drugs and a large volume of urine. In preliminary examination, the maximum water absorbing quantity of control sheet 1, 2 and HD safe sheet-U were 0.021, 0.078, and 1.059 g/cm^2^, respectively. Among the three sheets, HD safe sheet-U seemed to most rapidly absorb 1 mL of dropped artificial urine.

There was lower quantity of adhesion of most anticancer drugs from HD safe sheet-U to slipper soles compared with the two control sheets. For anticancer drug adhesion prevention performance of HD safe sheet-U, following three processes seemed to function: The scattered urine passes through a mesh sheet and is aspirated to an active carbon bed, followed by absorption of water layer through the capillary phenomenon. The urinary anticancer drug molecules coming in contact with active carbon during the aspiration process are adsorbed by active carbon. The anticancer drug once adsorbed by active carbon is isolated from slipper soles by using a mesh sheet. This mechanism of action of HD safe sheet-U was indicated in Fig. 4.

Active carbon is widely used for environmental clarification such as deodorization, waste water or gas disposal, and decoloration refinement of food. Moreover, active carbon has medical applications such as drug adsorption from gastrointestinal tract in cases of poisoning and for adsorption of uremic toxins in chronic renal failure [22, 23]. To our knowledge, apart from our previous report, no other study has utilized active carbon to adsorb an anticancer drug and to prevent diffusion. These results indicate that active carbon is effective in preventing diffusion of anticancer drug contamination through urine for the first time. For the clinical application of the HD safe sheet-U, we thought that a price should not disturb the popularity. An active carbon is relatively low-cost material, and the cost as HD safe sheet-U will be several hundred yen per 1 sheet (60 × 90 cm).

The present study had certain limitations. Difference in the adhesion to the slippers sole and adsorption by active carbon depending on the anticancer drug might be affect by the difference of wiped off recovery ratio and the determination limit. In Epi-ADR and VP-16, the lower recovery rate by the adsorption to cotton might lead the adhesion to the slippers bottom underestimated. Also, in CPA, IFM, and ADR, the determination limits were comparatively higher than others. This seemed to affect the interpretation of results. Tashiro et al investigated a cut-off of the environmental contamination of CPA, IFM and the GEM as 10 ng/mL [24]. Analysis with the determination limit of the ng/mL order using mass spectrograph is necessary for the future clinical evaluations. The validity of concentration and volume of the anticancer drug dropped on the sheets might need to be reconsidered in future studies. Urinary anticancer drug concentrations were calculated using dose, urinary excretion rate, and standard urine volume. Anticancer drug concentrations dropped on the sheet were higher than urinary concentrations considering the change in excretion rates and urine volumes. However, urinary excretion concentrations of anticancer drugs seem highly individual and are affected by dose, route of administration, and hepatic and renal functions of patients. Moreover, the urine concentrations at the time of maximum concentration (Tmax) may be higher than the mean concentrations calculated from an excretion rate for 24 h. Furthermore, depending on the use, situation, and lavatory shape, the urine volume to be scattered may be greater than the volume experimentally (1 mL) dropped on the sheet. Active metabolites may be included in the urinary excretion of an anticancer drug; for instance, active metabolites of CPA include 4-hydroxy cyclophosphamide and aldophosphamide, whereas those of IFM, MTX, CPT-11, and Epi-ADR include 4-hydroxy ifosfamide and aldoiphosphamide, 7-OH- methotrexate, SN-38, and epirubicinol, respectively. Active metabolites often have lower urinary concentrations compared with the unchanged drug, but these may have adverse effects on human exposure. We did not consider the adhesiveness of these urinary active metabolites in this study. This study was performed under experimentally controlled conditions. It was shown that HD safe sheet-U was superior in urinary anticancer drug adsorption characteristics as compared with the existing sheet in this condition. However, the final endpoint about the performance of the HD safe sheet-U seemed to be nonproliferation performance of environmental anticancer drug contamination and human exposure. Therefore, additional studies are warranted in the future to evaluate effects on environmental contamination and human exposure of anticancer drugs by using HD safe sheet-U in routine clinical practice. We had not yet determined at the exchange frequency of the HD safe sheet-U. Probably everyday exchange might be an ideal from hygienic perspective. However, it should be determined for the exchange cycle in consideration of maintenance of the adsorption performance by clinical evaluations.

## Conclusions

Active carbon might be able to adsorb the various anticancer drugs excreted in urine. Use of active carbon seems the unprecedented exposure-lowering method. HD safe sheet-U including active carbon might adsorb anticancer drugs from the scattered urine of cancer patients receiving chemotherapy, thus encloses contamination. Use of HD safe sheet-U might be effective in lowering anticancer drug exposure of healthcare workers and patients’ family members through urine including excreted anticancer drugs.

## Additional file

Additional file 1: Appendix 1 and 2. Adsorption properties of the activated carbon to the urinary anticancer drug. (ZIP 103 kb)

## Abbreviations

5-FU: 5-Fluorouracil
ADR: Doxorubicin
ANOVA: Analysis of variance
Ara-C: Cytarabine
BSC: Biological safety cabinet
CBDCA: Carboplatin
CDDP: Cisplatin
CPA: Cyclophosphamide
CPT-11: Irinotecan
DTX: Docetaxel
Epi-ADR: Epirubicin
GEM: Gemcitabine
IFM: Ifosfamide
MTX: Methotrexate
PPE: Personal protection equipment
PTX: Paclitaxel
VP-16: Etoposide
