# Supplementary material for: Development and evaluation of adsorption sheet (HD safe sheet-U) using active carbon for the purpose of the preventing the contamination diffusion of urinary excreted anticancer drug
Source: J Pharm Health Care Sci. 2017 Jun 2;3:16. doi: 10.1186/s40780-017-0085-8 (PMC5457606; doi:10.1186/s40780-017-0085-8)
Supplement: Additional file 1: — Appendix 1 and 2. Adsorption properties of the activated carbon to the urinary anticancer drug. (ZIP 103 kb) [file 40780_2017_85_MOESM1_ESM.zip › 2017.3.1 Appendix 1.pptx]

## Slide 1
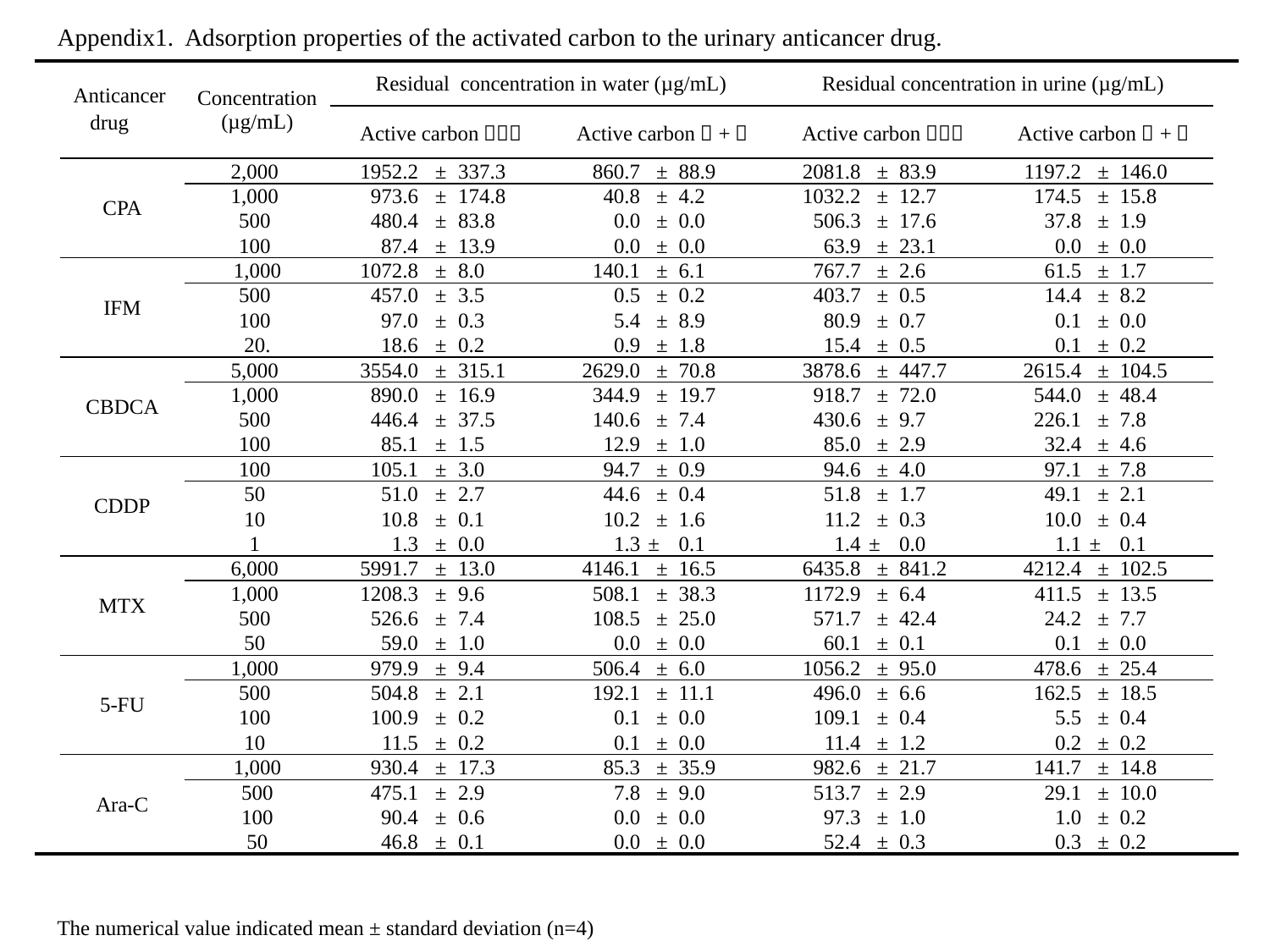

Appendix1. Adsorption properties of the activated carbon to the urinary anticancer drug.
| | Anticancer drug | Concentration (µg/mL) | Residual concentration in water (µg/mL) | | | | | | Residual concentration in urine (µg/mL) | | | | | | |
| --- | --- | --- | --- | --- | --- | --- | --- | --- | --- | --- | --- | --- | --- | --- | --- |
| | | | Active carbon（－） | | | Active carbon（+） | | | Active carbon（－） | | | Active carbon（+） | | | |
| | CPA | 2,000 | 1952.2 | ± | 337.3 | 860.7 | ± | 88.9 | 2081.8 | ± | 83.9 | 1197.2 | ± | 146.0 | |
| | | 1,000 | 973.6 | ± | 174.8 | 40.8 | ± | 4.2 | 1032.2 | ± | 12.7 | 174.5 | ± | 15.8 | |
| | | 500 | 480.4 | ± | 83.8 | 0.0 | ± | 0.0 | 506.3 | ± | 17.6 | 37.8 | ± | 1.9 | |
| | | 100 | 87.4 | ± | 13.9 | 0.0 | ± | 0.0 | 63.9 | ± | 23.1 | 0.0 | ± | 0.0 | |
| | IFM | 1,000 | 1072.8 | ± | 8.0 | 140.1 | ± | 6.1 | 767.7 | ± | 2.6 | 61.5 | ± | 1.7 | |
| | | 500 | 457.0 | ± | 3.5 | 0.5 | ± | 0.2 | 403.7 | ± | 0.5 | 14.4 | ± | 8.2 | |
| | | 100 | 97.0 | ± | 0.3 | 5.4 | ± | 8.9 | 80.9 | ± | 0.7 | 0.1 | ± | 0.0 | |
| | | 20. | 18.6 | ± | 0.2 | 0.9 | ± | 1.8 | 15.4 | ± | 0.5 | 0.1 | ± | 0.2 | |
| | CBDCA | 5,000 | 3554.0 | ± | 315.1 | 2629.0 | ± | 70.8 | 3878.6 | ± | 447.7 | 2615.4 | ± | 104.5 | |
| | | 1,000 | 890.0 | ± | 16.9 | 344.9 | ± | 19.7 | 918.7 | ± | 72.0 | 544.0 | ± | 48.4 | |
| | | 500 | 446.4 | ± | 37.5 | 140.6 | ± | 7.4 | 430.6 | ± | 9.7 | 226.1 | ± | 7.8 | |
| | | 100 | 85.1 | ± | 1.5 | 12.9 | ± | 1.0 | 85.0 | ± | 2.9 | 32.4 | ± | 4.6 | |
| | CDDP | 100 | 105.1 | ± | 3.0 | 94.7 | ± | 0.9 | 94.6 | ± | 4.0 | 97.1 | ± | 7.8 | |
| | | 50 | 51.0 | ± | 2.7 | 44.6 | ± | 0.4 | 51.8 | ± | 1.7 | 49.1 | ± | 2.1 | |
| | | 10 | 10.8 | ± | 0.1 | 10.2 | ± | 1.6 | 11.2 | ± | 0.3 | 10.0 | ± | 0.4 | |
| | | 1 | 1.3 | ± | 0.0 | 1.3 | ± | 0.1 | 1.4 | ± | 0.0 | 1.1 | ± | 0.1 | |
| | MTX | 6,000 | 5991.7 | ± | 13.0 | 4146.1 | ± | 16.5 | 6435.8 | ± | 841.2 | 4212.4 | ± | 102.5 | |
| | | 1,000 | 1208.3 | ± | 9.6 | 508.1 | ± | 38.3 | 1172.9 | ± | 6.4 | 411.5 | ± | 13.5 | |
| | | 500 | 526.6 | ± | 7.4 | 108.5 | ± | 25.0 | 571.7 | ± | 42.4 | 24.2 | ± | 7.7 | |
| | | 50 | 59.0 | ± | 1.0 | 0.0 | ± | 0.0 | 60.1 | ± | 0.1 | 0.1 | ± | 0.0 | |
| | 5-FU | 1,000 | 979.9 | ± | 9.4 | 506.4 | ± | 6.0 | 1056.2 | ± | 95.0 | 478.6 | ± | 25.4 | |
| | | 500 | 504.8 | ± | 2.1 | 192.1 | ± | 11.1 | 496.0 | ± | 6.6 | 162.5 | ± | 18.5 | |
| | | 100 | 100.9 | ± | 0.2 | 0.1 | ± | 0.0 | 109.1 | ± | 0.4 | 5.5 | ± | 0.4 | |
| | | 10 | 11.5 | ± | 0.2 | 0.1 | ± | 0.0 | 11.4 | ± | 1.2 | 0.2 | ± | 0.2 | |
| | Ara-C | 1,000 | 930.4 | ± | 17.3 | 85.3 | ± | 35.9 | 982.6 | ± | 21.7 | 141.7 | ± | 14.8 | |
| | | 500 | 475.1 | ± | 2.9 | 7.8 | ± | 9.0 | 513.7 | ± | 2.9 | 29.1 | ± | 10.0 | |
| | | 100 | 90.4 | ± | 0.6 | 0.0 | ± | 0.0 | 97.3 | ± | 1.0 | 1.0 | ± | 0.2 | |
| | | 50 | 46.8 | ± | 0.1 | 0.0 | ± | 0.0 | 52.4 | ± | 0.3 | 0.3 | ± | 0.2 | |
The numerical value indicated mean ± standard deviation (n=4)
