# Supplementary material for: Development and evaluation of adsorption sheet (HD safe sheet-U) using active carbon for the purpose of the preventing the contamination diffusion of urinary excreted anticancer drug
Source: J Pharm Health Care Sci. 2017 Jun 2;3:16. doi: 10.1186/s40780-017-0085-8 (PMC5457606; doi:10.1186/s40780-017-0085-8)
Supplement: Additional file 1: — Appendix 1 and 2. Adsorption properties of the activated carbon to the urinary anticancer drug. (ZIP 103 kb) [file 40780_2017_85_MOESM1_ESM.zip › 2017.3.1 Appendix 2.pptx]

## Slide 1
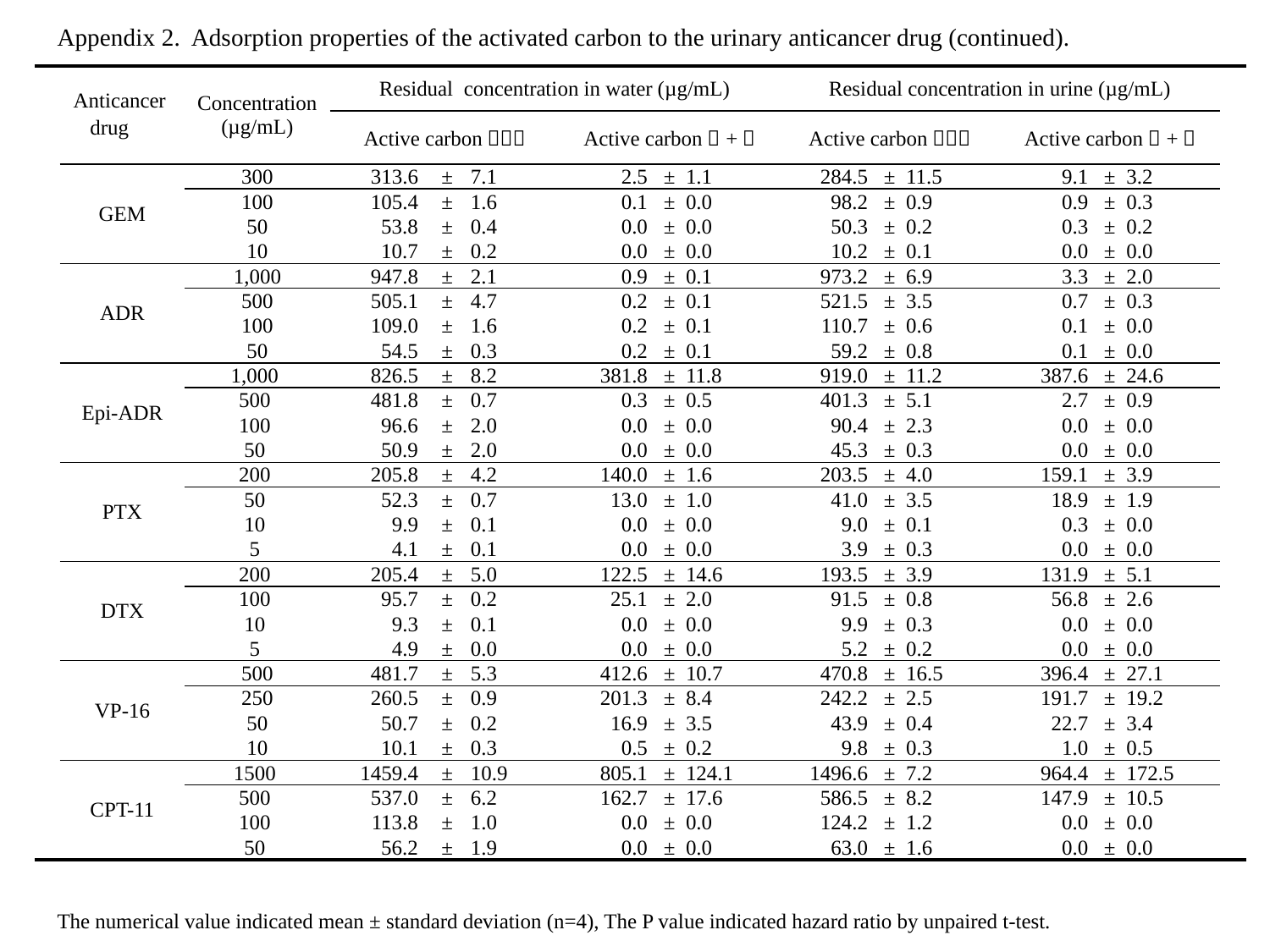

Appendix 2. Adsorption properties of the activated carbon to the urinary anticancer drug (continued).
| | Anticancer drug | Concentration (µg/mL) | Residual concentration in water (µg/mL) | | | | | | Residual concentration in urine (µg/mL) | | | | | | |
| --- | --- | --- | --- | --- | --- | --- | --- | --- | --- | --- | --- | --- | --- | --- | --- |
| | | | Active carbon（－） | | | Active carbon（+） | | | Active carbon（－） | | | Active carbon（+） | | | |
| | GEM | 300 | 313.6 | ± | 7.1 | 2.5 | ± | 1.1 | 284.5 | ± | 11.5 | 9.1 | ± | 3.2 | |
| | | 100 | 105.4 | ± | 1.6 | 0.1 | ± | 0.0 | 98.2 | ± | 0.9 | 0.9 | ± | 0.3 | |
| | | 50 | 53.8 | ± | 0.4 | 0.0 | ± | 0.0 | 50.3 | ± | 0.2 | 0.3 | ± | 0.2 | |
| | | 10 | 10.7 | ± | 0.2 | 0.0 | ± | 0.0 | 10.2 | ± | 0.1 | 0.0 | ± | 0.0 | |
| | ADR | 1,000 | 947.8 | ± | 2.1 | 0.9 | ± | 0.1 | 973.2 | ± | 6.9 | 3.3 | ± | 2.0 | |
| | | 500 | 505.1 | ± | 4.7 | 0.2 | ± | 0.1 | 521.5 | ± | 3.5 | 0.7 | ± | 0.3 | |
| | | 100 | 109.0 | ± | 1.6 | 0.2 | ± | 0.1 | 110.7 | ± | 0.6 | 0.1 | ± | 0.0 | |
| | | 50 | 54.5 | ± | 0.3 | 0.2 | ± | 0.1 | 59.2 | ± | 0.8 | 0.1 | ± | 0.0 | |
| | Epi-ADR | 1,000 | 826.5 | ± | 8.2 | 381.8 | ± | 11.8 | 919.0 | ± | 11.2 | 387.6 | ± | 24.6 | |
| | | 500 | 481.8 | ± | 0.7 | 0.3 | ± | 0.5 | 401.3 | ± | 5.1 | 2.7 | ± | 0.9 | |
| | | 100 | 96.6 | ± | 2.0 | 0.0 | ± | 0.0 | 90.4 | ± | 2.3 | 0.0 | ± | 0.0 | |
| | | 50 | 50.9 | ± | 2.0 | 0.0 | ± | 0.0 | 45.3 | ± | 0.3 | 0.0 | ± | 0.0 | |
| | PTX | 200 | 205.8 | ± | 4.2 | 140.0 | ± | 1.6 | 203.5 | ± | 4.0 | 159.1 | ± | 3.9 | |
| | | 50 | 52.3 | ± | 0.7 | 13.0 | ± | 1.0 | 41.0 | ± | 3.5 | 18.9 | ± | 1.9 | |
| | | 10 | 9.9 | ± | 0.1 | 0.0 | ± | 0.0 | 9.0 | ± | 0.1 | 0.3 | ± | 0.0 | |
| | | 5 | 4.1 | ± | 0.1 | 0.0 | ± | 0.0 | 3.9 | ± | 0.3 | 0.0 | ± | 0.0 | |
| | DTX | 200 | 205.4 | ± | 5.0 | 122.5 | ± | 14.6 | 193.5 | ± | 3.9 | 131.9 | ± | 5.1 | |
| | | 100 | 95.7 | ± | 0.2 | 25.1 | ± | 2.0 | 91.5 | ± | 0.8 | 56.8 | ± | 2.6 | |
| | | 10 | 9.3 | ± | 0.1 | 0.0 | ± | 0.0 | 9.9 | ± | 0.3 | 0.0 | ± | 0.0 | |
| | | 5 | 4.9 | ± | 0.0 | 0.0 | ± | 0.0 | 5.2 | ± | 0.2 | 0.0 | ± | 0.0 | |
| | VP-16 | 500 | 481.7 | ± | 5.3 | 412.6 | ± | 10.7 | 470.8 | ± | 16.5 | 396.4 | ± | 27.1 | |
| | | 250 | 260.5 | ± | 0.9 | 201.3 | ± | 8.4 | 242.2 | ± | 2.5 | 191.7 | ± | 19.2 | |
| | | 50 | 50.7 | ± | 0.2 | 16.9 | ± | 3.5 | 43.9 | ± | 0.4 | 22.7 | ± | 3.4 | |
| | | 10 | 10.1 | ± | 0.3 | 0.5 | ± | 0.2 | 9.8 | ± | 0.3 | 1.0 | ± | 0.5 | |
| | CPT-11 | 1500 | 1459.4 | ± | 10.9 | 805.1 | ± | 124.1 | 1496.6 | ± | 7.2 | 964.4 | ± | 172.5 | |
| | | 500 | 537.0 | ± | 6.2 | 162.7 | ± | 17.6 | 586.5 | ± | 8.2 | 147.9 | ± | 10.5 | |
| | | 100 | 113.8 | ± | 1.0 | 0.0 | ± | 0.0 | 124.2 | ± | 1.2 | 0.0 | ± | 0.0 | |
| | | 50 | 56.2 | ± | 1.9 | 0.0 | ± | 0.0 | 63.0 | ± | 1.6 | 0.0 | ± | 0.0 | |
The numerical value indicated mean ± standard deviation (n=4), The P value indicated hazard ratio by unpaired t-test.
